# Hormonal Treatment of Endometriosis: A Narrative Review

**DOI:** 10.3390/ph18040588

**Published:** 2025-04-17

**Authors:** Elvin Piriyev, Sven Schiermeier, Thomas Römer

**Affiliations:** 1 Chair of Gynecology and Obstetrics, University Witten-Herdecke, 58455 Witten, Germany; 2 Department of Obstetrics and Gynecology, Academic Hospital Cologne Weyertal, University of Cologne, Weyertal 76, 50931 Cologne, Germany; 3 Chair of Gynecology and Obstetrics, University of Cologne, 50923 Cologne, Germany

**Keywords:** endometriosis, hormonal treatment, combined oral contraceptive, progestins, GnRH agonists, GnRH antagonists, narrative review

## Abstract

**Background**: Endometriosis is one of the most common gynecological diseases, affecting up to 10–15% of women of reproductive age. It is a chronic, estrogen-dependent condition that often presents with heterogeneous symptoms, complicating diagnosis and delaying treatment. **Methods**: This is a narrative review based on a comprehensive analysis of recent literature regarding hormonal treatment options for endometriosis, including primary and adjuvant therapies. **Results**: Combined oral contraceptives (COCs) are effective in reducing dysmenorrhea, but show limited benefit for other symptoms and may not prevent disease progression. Progestins, particularly dienogest, demonstrate superior long-term efficacy with favorable side-effect profiles. GnRH agonists and antagonists are reserved for second-line treatment due to side effects and hypoestrogenism, but can significantly reduce endometriotic lesions. The levonorgestrel intrauterine system (LNG-IUS) is especially effective in patients with adenomyosis. **Conclusions**: Hormonal therapies are central to the management of endometriosis. Progestins are considered the most suitable long-term option. Despite promising results, evidence quality varies, and further studies are needed to establish long-term efficacy, patient-specific outcomes, and direct comparisons between agents.

## 1. Introduction

Endometriosis is a benign, estrogen-dependent disease that plays an important role in the reproductive phase of a woman’s life. The clinical presentation can also be very diverse, ranging from the patient being asymptomatic to experiencing invasive growth of endometriosis into neighboring organs. The heterogeneity in symptoms and localization complicates and delays diagnosis [1,2]. Endometriosis significantly affects patients’ quality of life, leading to chronic pelvic pain, fatigue, emotional distress, and sexual dysfunction. These symptoms often result in reduced productivity, absenteeism from work or school, and social withdrawal. Moreover, endometriosis is a major contributor to female infertility, affecting up to 30–50% of women with the condition. The disease also imposes a substantial economic burden, with high direct medical costs due to surgeries, repeated consultations, and long-term pharmacological treatments, as well as indirect costs from lost workdays and decreased work efficiency. Early diagnosis and effective management are therefore crucial not only for symptom control, but also for minimizing personal and societal costs [3]. The treatment of endometriosis consists of both medical and surgical approaches [1,2]. Individualized therapy planning—often a combination of both methods and, in some cases, including alternative treatments—is recommended. However, with the growing recognition of endometriosis as a chronic systemic disease, the importance of medical therapies is increasing [4,5]. After surgical treatment, adequate medical follow-up and regular monitoring to detect possible recurrence are essential [6]. Both primary and adjuvant hormonal therapy are key responsibilities for the outpatient gynecologist. In this context, knowledge of the diagnosis and treatment of endometriosis is indispensable for every gynecologist, given its high prevalence and the significant burden it places on affected patients. The aim of this narrative review is to provide an up-to-date overview of current hormonal treatment options for endometriosis, with a particular focus on the efficacy, safety, and clinical applicability of established therapies such as combined oral contraceptives and progestins, as well as newer agents including GnRH antagonists. In recent years, significant advancements have been made in understanding the long-term management of endometriosis and the role of individualized medical therapy. Given these developments, there is a critical need to synthesize the most recent literature to inform clinical decision-making and optimize treatment outcomes. This review responds to that need by summarizing and contextualizing the latest findings relevant to gynecological practice.

### 1.1. Epidemiology

Endometriosis is one of the most common diseases affecting women of reproductive age. The estimated prevalence is approximately 10–15% [7]. Placebo-controlled studies have shown that about 50% of endometriosis cases are progressive. Among patients with lower abdominal pain, the incidence ranges from 48% to 80%, and in those with infertility, between 20% and 48% [7]. Even in young women and adolescents with chronic lower abdominal pain unresponsive to standard pain therapy, the incidence can be as high as 70% [8]. In such cases, endometriosis is often not considered in the differential diagnosis until it is very late [8]. The average peak onset of the disease occurs around the age of 27 [9].

### 1.2. Definition and Classification

Endometriosis is defined as the presence of endometrium-like tissue (glands and stroma) outside the normal mucosal lining of the uterine cavity. This ectopic tissue undergoes cyclical changes similar to the endometrium and is associated with clinical symptoms and potentially destructive proliferative growth [1,2]. Endometriosis can be classified using the revised American Society for Reproductive Medicine (rASRM) classification system [10]. While the rASRM classification is widely used for staging endometriosis, it has notable limitations. It primarily focuses on the extent and location of superficial peritoneal lesions and adhesions, but it does not adequately reflect the severity of pain symptoms or the presence of deep infiltrating endometriosis (DIE). As a result, there is often a poor correlation between the rASRM stage and clinical symptoms, especially pain, which can be severe even in early-stage disease. Moreover, the system does not capture the full spectrum of disease manifestations, such as extragenital or bowel involvement, and it lacks guidance for surgical planning or prognosis. These limitations have led to the development and increasing use of complementary systems like the ENZIAN and #ENZIAN scores for better evaluation of DIE and surgical complexity [11]. Experts have established the ENZIAN score for the classification of rectovaginal endometriosis [1,7]. A new and useful combination of both classifications is the #ENZIAN score, which is becoming increasingly popular, especially in German-speaking countries [12]. Another important aspect is the differentiation between active and inactive endometriosis. In addition to the morphological appearance of endometriosis lesions—active lesions typically present as light papules and vesicles, whereas inactive lesions appear as scars or bluish nodules—it is also helpful to use proliferation markers, such as Ki-67, to support diagnosis and classification [13]. This differentiation can be clinically important for planning adjuvant hormonal therapy, as active endometriosis responds particularly well to hormonal treatments.

### 1.3. Symptoms

The symptoms are heterogeneous. The “4 Ds” of endometriosis (Dysmenorrhea, Dyspareunia, Dysuria, Dyschezia) summarize the most common symptoms [1,13,14,15]. While endometriosis is classically associated with cyclic pain patterns—such as dysmenorrhea and pain exacerbating during menstruation—non-cyclic pelvic pain is also a common presentation, particularly in advanced or deep infiltrating disease. This type of pain may persist throughout the menstrual cycle and significantly impacts quality of life, often mimicking symptoms of irritable bowel syndrome or interstitial cystitis. Additionally, patients may experience neuropathic pain, fatigue, and pain radiating to the legs or lower back, which are frequently underrecognized. Other less common symptoms include cyclical hematuria or rectal bleeding, suggesting urinary tract or bowel involvement, respectively. These atypical presentations underscore the importance of a high index of suspicion for endometriosis in patients with chronic, unexplained pelvic or systemic symptoms. Recurrent bleeding disorders may also occur. These symptoms should be specifically inquired about. The cyclical nature of the symptoms is particularly important for differential diagnosis (e.g., distinguishing from adhesions). Endometriosis is often only diagnosed during fertility evaluations [1,13,14,15].

### 1.4. Diagnostic

The symptoms often indicate the location of the endometriosis (Table 1). During the gynecological examination, a rectovaginal examination is also essential [1,14,16]. When adjusting the speculum (preferably a two-bladed speculum), the vagina should be assessed, as bluish or polypoid endometriosis lesions are often visible in the deeper areas [1]. Transvaginal sonography provides clues to possible changes in the ovaries (so-called chocolate cysts). Larger lesions of bladder endometriosis or rectovaginal nodules can also be diagnosed using transvaginal ultrasound. Transrectal sonography may be useful for assessing possible intestinal infiltration in cases of suspected rectovaginal endometriosis [1,2]. Laboratory tests currently have no definitive role in the diagnosis of endometriosis [17,18]. Although CA-125 is frequently used as a screening marker, its diagnostic value is limited due to low specificity and sensitivity. However, CA-125 is not a reliable diagnostic marker for endometriosis because it lacks both sensitivity and specificity. While CA-125 levels may be elevated in some patients with endometriosis, they can also be increased in a variety of other benign and malignant conditions, such as pelvic inflammatory disease, ovarian cysts, liver disease, and gynecologic cancers. This overlap significantly reduces its diagnostic accuracy, particularly in early-stage disease or in differentiating endometriosis from other causes of pelvic pain [1,2,15,17]. Other serological markers are not currently useful in clinical practice. This limitation contributes to the challenge of timely and accurate diagnosis of endometriosis. In Central Europe, an average of 7.7 years—and in some cases, up to 11 years—may pass between the first clinical symptoms and a confirmed diagnosis [9]. However, a reliable diagnosis of peritoneal endometriosis is still primarily made via laparoscopy. Other forms of endometriosis (ovarian, deep infiltrating, and adenomyosis) can now often be diagnosed through targeted clinical examination and imaging techniques, typically without the need for surgery [14].

## 2. Method

This article is a narrative review aiming to provide an updated overview of hormonal treatment options for endometriosis. A non-systematic, but comprehensive, literature search was conducted to identify relevant publications on the pharmacological management of endometriosis. The literature search was carried out in PubMed, Google Scholar, and Embase. Search terms included combinations of the following keywords: “endometriosis”, “hormonal treatment”, “combined oral contraceptives”, “progestins”, “dienogest”, “GnRH agonists”, “GnRH antagonists”. Due to the narrative nature of this review, no formal risk of bias assessment or meta-analysis was performed. Instead, the included studies were evaluated based on their clinical relevance and contribution to current therapeutic approaches. Key findings were synthesized to highlight current treatment strategies, advantages and disadvantages of each option, and clinical considerations for personalized therapy. This methodology aligns with the SANRA (Scale for the Assessment of Narrative Review Articles) criteria and reflects the aim of offering a concise but up-to-date summary of available evidence to guide clinical decision-making [19].

## 3. Treatment

### 3.1. Surgical Treatment

Nowadays, the surgical treatment of endometriosis should be used very selectively. It is crucial to carefully consider the appropriate timing. There are few absolute indications for surgery, such as intestinal obstruction, ureteral stenosis, and therapy-resistant pain after adequate hormonal treatment, or infertility. Subsequent hormonal therapy cannot compensate for insufficient surgical intervention [1,6,20]. When there is no absolute indication for surgery, primary hormonal therapy should be the first-line approach [1,2]. The indication for treatment—especially surgical intervention—should only be made in the presence of symptoms. Only the rare case of asymptomatic ureteral endometriosis, which can lead to hydronephrosis, warrants surgical treatment. In cases of extragenital endometriosis, complete surgical removal of all lesions should be performed in every case, due to limited or absent hormonal responsiveness [6,21]. The best time to perform surgery for this type of endometriosis is during menstruation, as it facilitates the identification of lesions, such as those in scar tissue, and enables more precise and thorough excision. In cases of isolated, completely excised extragenital endometriosis lesions, no hormonal follow-up therapy is necessary [6,21].

### 3.2. Hormonal Treatment

Hormonal therapy is the cornerstone of pharmacological treatment, as the disease is estrogen-dependent. The use of combined oral contraceptives (COCs) is primarily a symptomatic treatment for dysmenorrhea, especially when administered in extended-cycle or continuous regimens. There is extensive clinical experience with progestins, with the most robust and validated data available for dienogest. GnRH agonists are generally recommended only for short-term use due to their side-effect profile. For long-term management, an intrauterine system (IUS) releasing 52 mg of levonorgestrel (LNG) can also be used, particularly for rectovaginal endometriosis or adenomyosis [1,2,13,15]. Analgesics should be used only as an adjunct to causal (hormonal) therapies, as their efficacy is limited and not supported by strong evidence [22].

#### 3.2.1. Combined Oral Contraceptives

Combined oral contraceptives (COCs) are often used as the first-line therapy, either continuously for long-term use or in a long cycle (usually an 84/7-day regimen) [1,17,23]. This type of treatment is more effective than cyclical COC use [1,24,25,26]. Combined oral contraceptives (COCs) help alleviate dysmenorrhea primarily by suppressing ovulation and stabilizing hormone levels, which leads to a thinner endometrial lining. This reduction in endometrial tissue limits the amount of prostaglandins produced during menstruation. Prostaglandins are inflammatory mediators that cause uterine contractions and pain. By decreasing their production, COCs significantly reduce the intensity of menstrual cramps. Additionally, continuous or extended-cycle use of COCs can lead to amenorrhea, further minimizing cyclical pain symptoms in patients with endometriosis [27]. Hormonal contraceptives reduce the risk of retrograde menstruation and associated pelvic pain. Patients with endometriosis often appreciate the suppression of menstruation, particularly as it frequently results in amenorrhea. However, the use of combined oral contraceptives (COCs) in extended-cycle regimens has been proven effective primarily for postoperative follow-up after surgically treated endometriosis, or it may be used in patients with very mild symptoms who opt to avoid laparoscopy [25]. The vaginal ring demonstrates similar effectiveness to COCs, although it is less effective than progestin monotherapy [28]. While extended-cycle COCs are effective in managing dysmenorrhea, they are less effective for other symptoms such as dyspareunia [24,25,26]. Where relevant, contraindications to COC use—particularly those related to ethinylestradiol content—must be carefully considered [7]. The estrogen component of COCs is generally less favorable, as the beneficial effects of COCs in endometriosis appear to derive primarily from the progestin component [29]. Notably, studies have shown that patients who use COCs early for severe dysmenorrhea may have up to a 16-fold increased risk of developing deep infiltrating endometriosis later in life [30]. This suggests that the disease may progress even during COC use [29,30].

#### 3.2.2. Oral Progestins

Progestins have a well-established therapeutic effect on endometriosis and are suitable for the long-term treatment that is often required, primarily due to their favorable safety profile and low incidence of side effects [22,31,32,33]. Progestins exert their therapeutic effects on endometriosis by inducing decidualization and subsequent atrophy of both eutopic and ectopic endometrial tissue. They also suppress gonadotropin release, leading to a hypoestrogenic state, which limits the hormonal stimulation of endometrial implants. Furthermore, progestins have anti-inflammatory properties—they reduce local cytokine and prostaglandin production, which helps to alleviate pain and curb the inflammatory response associated with endometriotic lesions [27]. However, the data on most progestins in endometriosis therapy are relatively limited. To achieve the same effect as 2 mg of dienogest, most progestins require significantly higher (and often unapproved) doses (Table 2 and Table 3). Dienogest is a progestin available as a monotherapy for the treatment of endometriosis, which has also shown good efficacy in studies with regard to pain symptoms and has been found to be equally effective as GnRH analogues over a period of 6 months [34,35]. The effectiveness of dienogest was demonstrated in a placebo-controlled study, in which the pain score was significantly lower after 3 months compared to placebo [36]. The bleeding disorders that often occur initially are problematic, but these usually decrease significantly after approximately 3 months [7]. The main advantages over GnRH analogues include fewer side effects, lower costs, and the possibility of long-term use (Table 4) [32,37]. In contrast to desogestrel, 2 mg of dienogest is not approved for contraception, but it is highly contraceptive when used regularly. This dose is twice the ovulation-inhibiting amount (1 mg). With long-term use of dienogest, the combined progestogenic effects on the cervix and the endometrium contribute to its contraceptive properties [38]. There is now extensive data supporting the long-term use of dienogest in endometriosis treatment [34,38]. In ovarian endometriomas, dienogest therapy reduces the size of the lesions. After endometrioma surgery, the continuous use of 2 mg of dienogest significantly reduced the 5-year recurrence rate (4% vs. 69%) [39]. Extensive studies demonstrate that the postoperative use of dienogest reduces the recurrence rate to below 5% after endometrioma surgery [7]. In deep infiltrating endometriosis, the pain score is significantly reduced after 6 months, even in patients who were previously treated unsuccessfully with norethisterone acetate [40]. An intriguing approach is the vaginal administration of dienogest, although only a few case reports to date have documented a positive response [41]. The vaginal administration of dienogest is being explored as a potential alternative to oral delivery, based on the theoretical advantage of local drug delivery. Administering dienogest vaginally may lead to higher local concentrations at the pelvic sites where endometriotic lesions are commonly located, particularly in the posterior cul-de-sac, uterosacral ligaments, and rectovaginal septum. This targeted approach may enhance therapeutic efficacy while potentially reducing systemic side effects. Furthermore, vaginal delivery may bypass first-pass hepatic metabolism, offering improved bioavailability and a more consistent pharmacokinetic profile in some patients [41]. The matter requires further investigation. Particularly in cases of complicated surgery for deep infiltrating endometriosis, medical treatment options should be prioritized whenever possible [16]. Dienogest has also shown positive clinical results in adenomyosis, such as reductions in both pain and bleeding [42]. Dienogest is effective in all forms of endometriosis. Numerous studies have indicated that its side effect profile remains low, even with up to 5 years of continuous therapy. One study demonstrated that after 5 years of dienogest treatment, the average estradiol level of 28 pg/mL remained within the therapeutic window (20–50 pg/mL), thus minimizing the risk of bone mass loss [38]. In adolescents, however, a temporary reduction in bone density may occur with treatment durations exceeding one year, although this is typically followed by rapid recovery and consolidation [43]. The long-term clinical benefits of dienogest apply to patients receiving both primary and adjuvant hormone therapy [32,38]. Improvements in quality of life and sexual well-being can be expected from the long-term use of dienogest [44]. Depot progestins have also been shown to be effective in treating endometriosis, although their use is associated with a higher rate of side effects, including bone mass loss and an increased risk of thrombosis [1,2,10,15]. Depot progestins are long-acting formulations that provide continuous hormonal exposure, which is beneficial in managing endometriosis. A common example is medroxyprogesterone acetate (MPA), administered as an intramuscular injection. It effectively suppresses ovulation, induces endometrial atrophy, and alleviates endometriosis-associated pain. Another depot option includes norethisterone enanthate, though it is less commonly used. These formulations are particularly useful in patients who prefer less frequent dosing or have adherence challenges with daily oral medications [45]. The effectiveness of higher doses of oral MPA (30–50 mg/day) is lower compared to dienogest therapy [46]. Irregular bleeding, which can occur with all progestins, can be managed effectively depending on the timing and the endometrial thickness assessed via sonography (Table 5). An initial course of GnRH analogues for 2–3 months followed by a switch to 2 mg of dienogest can also reduce early bleeding; however, this approach does not enhance the treatment’s effectiveness in relieving pain [47].

#### 3.2.3. Local Progestins—Levonorgestrel Intrauterine System

Although the levonorgestrel-releasing intrauterine system (LNG-IUS) is not approved by the US Food and Drug Administration for the treatment of endometriosis-related pain, the use of intrauterine progestins (LNG-IUS) has proven to be effective for long-term therapy, particularly after surgical treatment of severe rectovaginal endometriosis [1,2,49]. The advantages include the minimal or non-existent systemic side effects of the progestins and the high local concentrations of levonorgestrel. The 52 mg of LNG-IUS is especially suitable for the treatment of adenomyosis and has been extensively studied. MRI-controlled studies have demonstrated a direct effect on adenomyosis lesions after 6 months [50]. LNG-IUS is more effective at controlling abnormal uterine bleeding caused by endometrial dysfunction than oral contraceptives, luteal-phase oral progestins, or nonsteroidal anti-inflammatory drugs [51]. In addition, one randomized clinical trial found no significant difference between LNG-IUS and the depot gonadorelin analog leuprorelin in reducing the visual analog scale (VAS) scores for endometriosis- and adenomyosis-related pain over 6 months of treatment [52].

#### 3.2.4. GnRH Agonist

GnRH agonists have been proven to be effective in the treatment of endometriosis for many years, but can only be used for a limited period of time. They work by downregulating pituitary GnRH receptors and inducing hypoestrogenism [53,54]. After a maximum period of 6 months, they must be discontinued or can only be used as long-term therapy in combination with add-back therapy (e.g., low-dose estrogens or tibolone) or administered intermittently. The use of GnRH analogues can be useful as follow-up therapy after severe endometriosis, as preparation for IVF/ICSI treatment, or as second-line therapy with add-back therapy in the case of inoperable, therapy-resistant severe findings [53,54].

#### 3.2.5. GnRH Antagonist Without Integrated Add-Back Therapy (Linzagolix)

GnRH antagonists work by directly and competitively binding to GnRH receptors in the anterior pituitary, thereby immediately blocking the release of gonadotropins (LH and FSH). This leads to a rapid suppression of ovarian estrogen production, which helps reduce the stimulation of endometriotic lesions and alleviates associated symptoms. In contrast to GnRH agonists, antagonists do not cause an initial hormonal flare, making them a more favorable option in certain clinical settings [55]. A recent study (EDELWEISS 3) [56] investigated the effectiveness of linzagolix. The EDELWEISS Phase 3 study was conducted at 91 clinical centers across the USA (*n* = 26) and Europe (*n* = 65). It was a prospective, randomized, placebo-controlled, double-blind, double-dummy trial. The study included a 3-month screening period, a 6-month treatment phase, and a 6-month drug-free follow-up period. Participants were randomized in a 1:1:1 ratio into one of three groups: placebo, 75 mg linzagolix alone, or 200 mg linzagolix plus add-back therapy (ABT) (1.0 mg E2/0.5 mg NETA). Treatment was administered orally once daily for up to 6 months. Treatment response was rapid. Both linzagolix doses resulted in significant reductions in dysmenorrhea and non-menstrual pelvic pain compared to placebo after just 1 to 2 months of treatment. The 200 mg daily dose with ABT met the primary efficacy endpoint, showing clinically meaningful improvements in both dysmenorrhea and non-menstrual pelvic pain after 3 months, with no increase—or even a decrease—in analgesic use. The estimated responder rate for dysmenorrhea in the 200 mg linzagolix + ABT group was 72.9%, compared to 23.5% in the placebo group. At 3 months, the 75 mg linzagolix dose without ABT also showed a clinically meaningful and statistically significant reduction in dysmenorrhea compared to placebo. The responder rate was 44.0% in the 75 mg group versus 23.5% in the placebo group. Although the 75 mg dose showed a trend toward reducing non-menstrual pelvic pain at 3 months, the difference was not statistically significant (*p* = 0.279) [56].

It was found that after 6 months, the group receiving 200 mg of linzagolix with ABT experienced significantly less dysmenorrhea (*p* < 0.001) and significantly less non-menstrual pelvic pain (*p* < 0.001) compared to the placebo group. The 75 mg linzagolix group also showed significant improvements over the placebo in both dysmenorrhea (*p* = 0.036) and non-menstrual pelvic pain (*p* = 0.003) at 6 months. Statistically significant reductions in dyschezia, overall pelvic pain, and limitations in daily activities were observed in the 200 mg linzagolix with the ABT group compared to the placebo group. Similarly, though generally smaller, improvements in these secondary endpoints were also noted with the 75 mg dose. The only exception was dyschezia, where both linzagolix doses showed equal effectiveness [56].

In conclusion, a combination of 200 mg linzagolix, estradiol, and norethisterone acetate was found to significantly reduce endometriosis-associated pain and improve quality of life, while minimizing the risks of bone loss and vasomotor symptoms, due to the add-back therapy. At 3 months, a daily dose of 75 mg linzagolix significantly improved dysmenorrhea. By 6 months, it showed benefits for both dysmenorrhea and non-menstrual pelvic pain. There was minimal bone loss, as serum estradiol levels were only partially suppressed [56].

#### 3.2.6. GnRH Antagonist with Integrated Add-Back Therapy (Relugolix-CT)

Monotherapy with a GnRH agonist or antagonist has only suboptimal efficacy at low doses and causes undesirable hypoestrogenic side effects, such as hot flashes and bone density loss, at higher doses. To address these issues, relugolix-CT was developed as an oral combination therapy to minimize vasomotor symptoms and prevent significant bone loss while maintaining efficacy [57]. As part of the SPIRIT study program, 40 mg of relugolix, 1 mg of estradiol (as hemihydrate), and 0.5 mg of norethisterone acetate were evaluated over 104 weeks for efficacy and safety. This evaluation included two replicate studies (SPIRIT 1 and SPIRIT 2) and a subsequent long-term extension study. SPIRITS 1 and 2 were randomized, double-blind, placebo-controlled trials lasting 24 weeks, conducted in premenopausal women (18–50 years) who had received an endometriosis diagnosis within the past 10 years through surgery, direct visual evidence, or histological confirmation. Only patients with moderate to severe dysmenorrhea and non-menstrual pelvic pain were included. SPIRIT 1 enrolled 638 patients, and SPIRIT 2 enrolled 623 [48]. The percentage of responders from baseline to the end of the 24-week study in the relugolix-CT group was 75% for dysmenorrhea in both studies, 59% for non-menstrual pelvic pain in SPIRIT 1, and 66% in SPIRIT 2 [48]. At week 52, among patients who had received continuous relugolix-CT treatment, 85% responded for dysmenorrhea and 74% for non-menstrual pelvic pain. These response rates were maintained through week 104: 85% for dysmenorrhea and 76% for non-menstrual pelvic pain [58]. Amenorrhea occurred in 57.7% (SPIRIT 1) and 58.5% (SPIRIT 2) of women during the final 11 weeks of the 24-week treatment period. Furthermore, the relugolix-CT group maintained a consistent 59% reduction in dyspareunia through week 104 compared to baseline. The amenorrhea rate increased throughout the treatment, reaching 79.6% at week 52 and 76.9% at week 104 [48,58]. Bone mineral density (BMD) at the lumbar spine and hip decreased by less than 1% in the relugolix-CT arm after 24 weeks. Following this initial decrease, BMD plateaued at week 36 and remained stable throughout the 104-week treatment period. Relugolix-CT significantly improved menstrual and non-menstrual pain, dyspareunia, and pain-related disability in women with endometriosis for up to two years. Importantly, after the initial decline of <1% in BMD, bone density remained stable with continued treatment [48,58]. According to the manufacturer, relugolix-CT treatment is not time-limited. Long-term safety data are currently available for up to two years of continuous use. Treatment can be discontinued upon the onset of menopause.

## 4. Recurrence and Prognosis

The recurrence rate of endometriosis varies widely, ranging from 10% to 80%, depending primarily on the extent and biological activity of the disease. A key prognostic factor for recurrence is the radicality of the initial surgical intervention, as well as the implementation of appropriate hormonal follow-up therapy. Radical surgery in endometriosis aims to remove all visible endometriotic lesions, which can significantly alleviate symptoms and improve fertility outcomes. However, due to the disease’s infiltrative and microscopic nature, complete excision of all endometriotic tissues is often not possible. Therefore, postoperative hormonal therapy is essential to suppress any residual microscopic disease, reduce the risk of recurrence, and maintain long-term symptom relief. This combined approach offers a more comprehensive and sustainable management strategy for patients with moderate to severe endometriosis [17]. Endometriosis often presents with complex symptoms that impact multiple aspects of a woman’s health, including pelvic pain, infertility, gastrointestinal or urological symptoms, and psychological distress. As such, optimal management frequently requires a multidisciplinary approach involving gynecologists, reproductive endocrinologists, pain specialists, colorectal and urologic surgeons, and mental health professionals. This collaboration ensures comprehensive, individualized care, particularly for patients with deep infiltrating endometriosis or those seeking fertility preservation or restoration. Reproductive specialists play a pivotal role in evaluating and optimizing fertility outcomes, guiding hormonal therapy selection, and planning for assisted reproductive technologies when necessary [59]. Although endometriosis cannot be completely cured, long recurrence-free intervals can be achieved—even in cases of severe disease—through the optimal coordination of all available treatment options, particularly surgical and pharmacological therapies. Progestogens, especially dienogest, play a central role in long-term management, as supported by the most robust clinical data (Table 6) [38,39]. The decision to initiate adjuvant hormonal therapy should primarily consider the location, activity, and symptomatology of the endometriosis. In patients with fertility desires, reproductive medical interventions can often help fulfill this goal. For postmenopausal women, including those who have undergone hysterectomy and have a history of severe endometriosis, tibolone or a combination of estrogen and progestogen is preferred if hormone replacement therapy is indicated. The use of estrogen-only HRT after menopause is associated with a potential risk of reactivating residual or microscopic endometriotic lesions. Estrogen stimulates the proliferation of endometrial-like tissue, which can lead to recurrence of symptoms or even malignant transformation in rare cases. Therefore, combined HRT (estrogen plus a progestin) is generally recommended for these patients, as progestins can counteract the proliferative effects of estrogen on ectopic endometrial tissue [60,61].

## 5. Conclusions

Endometriosis is a common and often debilitating disease affecting women during their reproductive years. Early diagnosis improves treatment outcomes and overall prognosis. Both hormonal and surgical interventions are valid treatment options and should be tailored to the individual based on symptom severity, fertility desires, and clinical findings. In cases of extensive disease involving adjacent organs, interdisciplinary management in a specialized endometriosis center is essential. While numerous hormonal therapies—such as combined oral contraceptives, oral and local progestins, and GnRH analogues and antagonists—have demonstrated clinical efficacy, the quality of evidence varies. Some treatments are supported by large randomized controlled trials (e.g., relugolix-CT and linzagolix), while others (vaginal dienogest, LNG-IUS) rely on observational data or smaller studies. Furthermore, direct head-to-head comparisons between different agents are scarce, and long-term safety data—particularly regarding bone mineral density, metabolic effects, and fertility preservation—are limited for several newer therapies. These limitations highlight the need for future research involving well-designed, long-term studies that compare different hormonal regimens directly and explore their efficacy in diverse subpopulations. Attention to the impact of treatment on quality of life, recurrence prevention, and individualized care strategies remains essential for advancing endometriosis management.

## 6. Summary

- Endometriosis should also be considered in adolescents presenting with lower abdominal pain and dysmenorrhea. Early diagnosis and timely initiation of treatment are crucial for preventing the chronic progression of the disease.
- Primary hormonal therapy, even in the absence of histological confirmation, is currently the most important treatment approach. Surgical intervention should be reserved for clearly defined indications, such as endometriotic cysts or organ obstruction.
- Adjuvant hormonal therapy is essential following surgical management to reduce recurrence risk.
- Progestins are the first-line pharmacological treatment. It is important to consider differences in efficacy, regulatory approval, and side-effect profiles. Among these, dienogest is the most thoroughly studied and has demonstrated the highest efficacy in endometriosis management, making it particularly suitable for long-term use.
- GnRH antagonists, in combination with add-back therapy, have become established as an effective therapeutic option for endometriosis.
- Hormonal adjuvant therapies may be continued in the long term, provided they are effective and well tolerated.
- In cases involving adenomyosis, specific considerations are necessary. A 52 mg levonorgestrel-releasing intrauterine system (LNG-IUS) may be considered as the first-line therapy in these patients.

Table 7 summarizes the hormonal treatments for endometriosis.

## Figures and Tables

**Table 1:** Symptoms and localization of endometriosis [7,13].

Symptoms	Localization
Dysmenorrhea Dyspareunia/Dyschezia Recurrent Cystitis/Dysuria Cyclic scar pain Cyclic shoulder pain Hematuria, cyclic bladder symptoms Hematochezia, Dyschezia	Peritoneal endometriosis Rectovaginal endometriosis Bladder peritoneum Scar Diaphragma Bladder (DIE) Bowel (DIE)

DIE—deep infiltrated endometriosis.

**Table 2:** Transformation doses of various progestins in comparison [7,38].

Progestin	Transformation Dose (mg/Cycle)	Available Dose (mg/Tablet)	Recommended Dose (mg/Day)	Number of Tablets per Day
Dienogest (DNG)	6	2	2	1
Desogestrel (DSG) *	2	0.075	0.2–0.4	3–5
Chlormadinonacetat (CMA) *	25	2	4–8	2–4
Medroxprogesteronacetat (MPA) *	50	5	20–100	4–20
Dydrogesteron (DYD) *	140	10	20–60	2–6

* off-label-use.

**Table 3:** Progestins for the endometriosis treatment [7,38].

	Dienogest	Other Oral Progestins (DSG, CMA, MPA, DYD)
Admission	Yes	No
Required dosage	2 mg/day	High dose (2–20 times the usual dose)
Progestin-related side effects at the required dosage	Low	often
Direct effect on endometriosis lesions	proven	not proven
Influence on bone density	No (only <18 years old)	Yes (at higher/required dosage)
Placebo-controlled studies	available	Not available
Long-term data	available	Not available

DSG: desogestrel; CMA: chlormadinonacetat; MPA: medroxprogesteronacetat; DYD: dydrogesteron.

**Table 4:** Advantages and disadvantages of medical treatments for endometriosis [7,38,48].

	Hormonal Contraceptives	Progestins	GnRH Agonists	GnRH Antagonists with Add-Back Therapy
Contraception	+++	++	++	++
Cycle stability	++	+	+++	++
Risks (e.g., thrombosis)	++	+	+	+
Long-term use	++	+++	+	+++
Costs	+	+/++	+++	+++
Side effects	+	+	+++	+
Approved indications	-	+++ (Dienogest)	+++	+++

+ low; ++ middle; +++ high.

**Table 5:** Management of bleeding disorders in the application of 2 mg of dienogest [38].

Initial bleeding disorder (<3 months)	Bleeding disorder during the therapy (>6 months)	
↓	↓	
Double endometrial thickness usually > 5 mm	Double endometrial thickness	
↓		
4 mg of DNG for the first 8 weeks or GnRH analogue for 2–3 months	usually <5 mm	seldom >5 mm
	Break for 7 days or 1 mg of estradiol for 7 days	DNG 4 mg for 4 weeks

DNG—dienogest.

**Table 6:** Possibilities of long-term medication therapy (>1 year) for endometriosis [7,38,48].

Combined hormonal contraceptives; Progestins: systemic (oral, injection); Local (LNG-IUS); GnRH agonists: continuously (+add-back therapy)/intermittent; GnRH antagonists with add-back therapy.

**Table 7:** Summary table of hormonal therapies for endometriosis.

Agent	Indication	Mechanism of Action	Dose/Regimen	Efficacy	Advantages	Limitation	Common Side Effects
Combined Oral Contraceptives (COCs) [1,7,17,23,24,25,26,27,28,29,30]	First-line therapy for pain symptoms (esp. dysmenorrhea); not curative	Suppress ovulation; reduce endometrial proliferation	Continuous or cyclic use (84/7-day regimen) (e.g., ethinyl estradiol 20–35 μg + progestin)	Effective for dysmenorrhea; less effect on deep infiltrating lesions	Suppress bleeding; accessible; used post-surgery or in mild cases	Estrogen content may support disease progression; associated with increased risk of deep infiltrating endometriosis (DIE) with early use; not ideal for all symptoms	Nausea; breast tenderness; thromboembolism risk; disease progression is possible
Oral Progestins (e.g., dienogest) [7,22,31,32,33,34,35,36,37,38,39,40,41,42,43,44]	First-line long-term therapy for pain and suppression	Induce decidualization and atrophy of endometrial tissue	Dienogest 2 mg daily	High efficacy for pain reduction and lesion suppression (esp. superficial and deep lesions)	Low side effects; long-term use possible; effective in all types; positive QoL effects	Initial bleeding irregularities; not approved as a contraceptive (though effective); temporary BMD reduction in adolescents	Initially irregular bleeding; headache; mood changes; decreased libido
Depot Progestins (e.g., MPA) [1,2,10,46]	Alternative to oral progestins; suitable for non-compliant patients	Induce decidualization and atrophy of endometrial tissue	MPA 150 mg IM every 3 months	Effective for pain relief and lesion reduction	Low side effects; long-term use possible; effective in all types; positive QoL effects	Higher rate of side effects (bone loss, thrombosis); bleeding issues	Weight gain; bone density loss; irregular bleeding; delayed return of fertility
LNG-IUS (Levonorgestrel 52 mg IUD) [1,2,49,50,51,52]	Preferred in adenomyosis or for long-term localized suppression	Local progestogenic effect → decidualization and atrophy of the endometrial lining and decrease in pro-inflammatory cytokines and prostaglandins	Releases 20 µg/day of levonorgestrel; replaced every 5 years	Reduces dysmenorrhea and heavy bleeding; local suppression of endometrial implants	Minimal systemic effects; good for long-term use; stops abnormal bleeding	Not FDA-approved for endometriosis	Initially irregular bleeding; ovarian cysts; expulsion
GnRH Agonists (e.g., Leuprolide) [53,54]	Second-line therapy for refractory cases or short-term use	Downregulate pituitary GnRH receptors → hypoestrogenism	Leuprolide acetate 3.75 mg IM monthly	Reduce pain and lesion size; induce hypoestrogenism	Useful for short-term or pre-IVF/ICSI	Hypoestrogenic side effects (BMD loss, vasomotor symptoms); require add-back therapy for long-term use	Hot flashes; bone loss; mood changes; require add-back therapy
GnRH Antagonists (Linzagolix) [56]	Second-line therapy for moderate-to-severe pain	Immediate receptor blockade → reduced estrogen without flare effect	75 mg daily or 200 mg + ABT	Rapid suppression of estrogen; effective for pain; good tolerability	Oral administration; effective for dysmenorrhea and pelvic pain; improve QoL	75 mg less effective for non-menstrual pain; higher doses (200 mg) require ABT	Hot flashes; headache; bone loss (dose-dependent); less flare-up than agonists
GnRH Antagonists with ABT (Relugolix–CT) [48,57,58]	Second-line therapy for moderate-to-severe pain	Immediate receptor blockade → reduced estrogen without flare effect	Relugolix-CT 40 mg daily (with add-back therapy)	Long-term improvement in dysmenorrhea and pelvic pain; high responder rate (up to 85%)	Long-term use possible (104 weeks); minimal BMD loss (<1%); well-tolerated up to 2 years; reduce dyspareunia and amenorrhea rates	Require continuous therapy; cost and access may vary	Hot flashes; headache
Vaginal Dienogest (Investigational/Off-label) [41]	Theoretical benefit in localized lesions; not yet standard	Higher local concentrations; induces decidualization and atrophy of endometrial tissue	2 mg vaginally (experimental use)	Potential for higher local concentration; lower systemic side effects	Higher local concentrations at the pelvic sites where endometriotic lesions are commonly located; potentially reduces systemic side effects; may bypass first-pass hepatic metabolism; offers improved bioavailability and a more consistent pharmacokinetic profile	Not well established; further studies are needed	Not well established; further studies are needed

## Data Availability

Data are contained within the article.
